# Photopatternable PEDOT:PSS/PEG hybrid thin film with moisture stability and sensitivity

**DOI:** 10.1038/micronano.2017.4

**Published:** 2017-04-10

**Authors:** Zijie Zhu, Gaomai Yang, Ruya Li, Tingrui Pan

**Affiliations:** 1 Micro-Nano Innovations (MiNI) Laboratory, Department of Biomedical Engineering, The University of California, Davis, CA 95616, USA; 2 Department of Electrical and Computer Engineering, The University of California, Davis, CA 95616, USA

**Keywords:** breath rate measurement, humidity sensor, hybrid thin film, moisture stability, one-step photopatterning, PEDOT:PSS, perspiration tracking

## Abstract

Degradation and delamination resulting from environmental humidity have been technically challenging for poly (3,4-ethylenedioxythiophene): poly(styrene sulfonate) (PEDOT:PSS) thin-film processing. To overcome this problem, we introduced a one-step photolithographic method to both pattern and link a PEDOT:PSS film onto a poly (ethylene glycol) (PEG) layer as a hybrid thin film structure on a flexible substrate. This film exhibited excellent long-term moisture stability (more than 10 days) and lithographic resolution (as low as 2 μm). Mechanical characterizations were performed, including both stretching and bending tests, which illustrated the strong adhesion present between the PEDOT:PSS and PEG layers as well as between the hybrid thin film and substrate. Moreover, the hybrid moisture-absorbable film showed a quick response of its permittivity to environmental humidity variations, in which the patterned PEDOT:PSS layer served as an electrode and the PEG layer as a moisture-sensing element. Perspiration tracking over various parts of the body surface as well as breath rate measurement under the nose were successfully carried out as demonstrations, which illustrated the potential utility of this stable hybrid thin film for emerging flexible and wearable electronic applications.

## Introduction

Conducting polymers (CPs) have attracted increasing attention over past decades due to their unique combinations of chemical and physical properties from metallic and polymeric materials, including mobile charged species and a flexible and tunable matrix^1–12^. Compared with carbon-based nanomaterials, for example, carbon nanotubes, graphene, and nanowires, CPs offer higher flexibility and greater tunability in the material properties^3,4^. In particular, as a highly researched CP, poly (3,4-ethylenedioxythiophene): poly(styrene sulfonate) (PEDOT:PSS) has been recently extended to various applications, such as optoelectronics^5^, thin-film transistors^6^, transparent electrodes^7^, super-capacitors^8^, surfaces for cell cultures^9^, tissue engineering^10^, biosignal recording^11^, and solar cells^12^, due to its high conductivity, optical transparency, mechanical flexibility, excellent biocompatibility, and low-cost manufacturability. To establish an aqueous dispersion of PEDOT, PSS is introduced to balance the positively charged PEDOT backbone. From this dispersion, PEDOT:PSS can be spin coated easily to form a conductive thin film on planar surfaces^13^.

However, similar to other conductive organic thin films, degradation and delamination of PEDOT:PSS thin films under aqueous or humid environments can be still very challenging^14–22^, although many studies have been attempted to address the moisture-stability issue. For example, PEDOT:PSS stability has been reportedly improved by adding different synthetic reagents, such as ionic liquids^15^, Nafion^16^, poly (ethylene glycol) (PEG) methyl ether^17^, formic acid^18^, and bis (fluorinated phenyl azide)^19^. However, long-term water stability was not reported in these studies. Alternatively, by adding poly (vinyl alcohol) and multi-walled carbon nanotubes^20^ or biopolymer sodium carboxymethyl cellulose^21^, PEDOT:PSS films can be stabilized in a moist environment or even in water for several days. However, the new composition leads to relatively low electrical conductivity (<10 S cm^−1^) and poor optical transparency of the thin film. Therefore, it remains a technical challenge to achieve a PEDOT:PSS thin film with humidity stability while retaining its high electrical conductivity and optical transparency^22^.

In addition to its mechanical flexibility and the tunability of the material properties, photopatternability of CPs can be a highly attractive feature in various emerging applications, including optoelectronics^23^, photovoltaics^24^, and medical devices and sensors^25^, where the desired microscopic definitions and resolutions can determine the overall device performance and the simplified process flow leads to a reduction of the manufacturing cost and improvement of the product yield^26^. From a manufacturing perspective, it is essential to establish the least complicated process flow to form micropatterns of CPs without any potential modification of the desired conductivity and surface morphology. Printing (ink-jet printing^27^ and roll-to-roll printing^28^), lithography (photolithography^29^, nanoimprint lithography^30^, UV and e-beam lithography^31^), and direct etching (laser ablation^32^ and plasma etching^33^) are presently the mainstream micropatterning techniques for CPs. Among them, photolithography has attracted the most attention because of its low cost, high resolution, compatibility with the established manufacturing process, and large surface area patternability^29^. Unfortunately, currently, the traditional photolithography technique has not yet been made compatible with PEDOT:PSS processing. This is primarily because the PEDOT:PSS film is unstable in most developer solutions, and the typical photoresists used in photolithography are acid sensitive, which can be chemically affected by the acidic PEDOT:PSS film^34^. As an alternative, a protection layer has been used and reported in the photolithographic process to pattern a PEDOT:PSS film, which requires additional deposition as well as selective etching and removal, leading to increased processing costs and post-processing complications^35^.

In this communication, we have first reported a simple method to fabricate a photopatternable and highly conductive PEDOT:PSS/PEG hybrid thin film with exceptional moisture stability and sensitivity on flexible substrates. Compared with the current photolithographic methods for PEDOT:PSS processing, our photopatternable PEDOT:PSS/PEG approach is rather straightforward and simple; by assembling a photomask, an activated polydimethylsiloxane (PDMS) film, a photosensitive poly (ethylene glycol) diacrylate (PEGDA) layer, and a PEDOT:PSS-coated glass and using UV exposure, the process avoids using any photoresist or protection layer. Unlike the direct coating of the PEDOT:PSS film on a surface that can be easily damaged by moisture^14^, the obtained hybrid film can be highly moisture-stable, benefiting from the flexible matrix of the PEG layer^36^. Moreover, the conductivity of the film does not show a substantial change (<10%) after being submerged in deionized (DI) water for 10 days. In addition, this hybrid film exhibits excellent flexibility and stretchability under mechanical loads. After extended cyclic mechanical loads (over 1000 cycles), the electrical conductivity remains almost unaltered (with a change of <0.1%), while after 500 cycles of the 10% strain test, the conductivity varies within 10% range. To further investigate its utility, a wearable humidity sensor was constructed in a capacitive sensing mode using the patterned PEDOT:PSS/PEG thin film. In such a case, the PEDOT:PSS layer provides long-term stability and high conductivity as the electrode and the PEG matrix is not only employed as the crosslinking substrate but also as a responsive material affected by environmental moisture change, as its permittivity varies due to vapor condensation and moisture diffusion in its nanoporous structure. To tune the moisture sensitivity of the device, different pore sizes and thicknesses of the hybrid PEDOT:PSS/PEG thin films have been fabricated and characterized. A series of moisture detection cases have been presented, including environment humidity monitoring, characterization of humidity gradients, and wearable tracking of perspiration and breath rates.

## Materials and methods

### Materials

PEDOT:PSS solutions (Clevios PH 1000) were purchased from Heraeus, Hanau, Germany, which had a PEDOT:PSS ratio of 1:2.5. Acetone and ethylene glycol from Fisher Scientific, Fair Lawn, NJ, USA; isopropyl alcohol, methanol, and ethanol from EMD Millipore, Billerica, MA, USA; PEGDA-700, benzophenone, sodium periodate (⩾99.8%), and benzyl alcohol (anhydrous, 99.8%) from Sigma-Aldrich, St. Louis, MO, USA, were used as received. The mass ratio of base to curing agent of PDMS (Sylgard 184, Dow Corning, Auburn, MI, USA) was chosen to be 10:1. The prepolymers were mixed and then cured at 120 °C for 30 min. A commercial PEDOT:PSS conducting film (Kodak, Rochester, NY, USA) was used for comparison^37^.

### Preparation of PEDOT:PSS/PEG hybrid films on PDMS

In a standard procedure for PEDOT:PSS film preparation^38^, the PEDOT:PSS solution was first filtered using 0.45 μm syringe filters and then spin coated on clean glass substrates at 5000 rpm for 30 s (WS-400-6NPP, Laurell, North Wales, PA, USA). The glass substrates were all rinsed by acetone and isopropyl alcohol in sequence before use. After baking at 120 °C on a hot plate in ambient air for 15 min, PEDOT:PSS-coated glass substrates were immediately immersed in an ethylene glycol bath for 15 min and then annealed for another 15 min at 120 °C. Multilayer PEDOT:PSS films on glass were obtained by repeating the previous steps. PDMS slabs were photoactivated by being immersed in a 10 wt% benzophenone solution in acetone for 2 min and then rinsed by methanol three times. One milliliter of 40 wt% PEGDA solution was prepared by mixing 400 μL of PEGDA-700, 10 μL of 100 mM NaIO_4_, and 50 μL of 5 wt% benzyl alcohol in DI water. Then, both the PEDOT:PSS-coated glass and the PDMS slabs were treated with oxygen plasma (FEMTO, Diener, Ebhausen, Germany) for 30 s at 90 W to increase the surface hydrophilicity. In this way, the PEGDA-containing solution can be easily introduced and spread between the PDMS and glass substrates. To control and obtain different thicknesses of the PEG layer, we have added specific volumes of PEGDA-containing solution to wet the same substrate area. PEGDA was photopolymerized under UV exposure (365 nm, ABM, Scotts Valley, CA, USA) with intensity of 20 mW cm^−2^ for 5 min through specific photomasks, and then the glass substrate was removed with PEDOT:PSS linked onto the polymerized PEG matrix. As a result, specific photopatterns were developed by the direct photolithographic approach, followed by rinsing away unreactive PEGDA residues with DI water.

### Characterizations of PEDOT:PSS/PEG hybrid films on PDMS

To observe PEDOT:PSS patterning with the one-step photolithographic method, optical microscopic images were taken using an EVOS XL transmitted-light microscope (Thermo Fisher Scientific, Lafayette, CO, USA). To test the moisture stability of the hybrid films, samples were submerged in DI water or sonicated by using an ultrasonic bath from Thermo Fisher Scientific. To demonstrate optical transmittance changes before and after moisture exposure, a microplate reader (Tecan Safire2, Männedorf, Switzerland) was used. Each sample was tested on three positions with visible light wavelength ranging from 380 to 780 nm. To characterize the mechanical properties of the hybrid film, a computer-controlled step motor (VT-80, PI Micos, Freiburger, Germany) with a spatial resolution of 400 nm was used to precisely control the loads. We have applied electrically conductive double sided tape (3M-9703, 3M, St. Paul, MN, USA) to establish connections from the PEDOT:PSS film to a custom readout circuit with patterned copper traces at a sampling frequency of 1 kHz. An FEI 430 Nano SEM (FEI, Hillsboro, OR, USA) was used to facilitate the observation of morphology changes that occurred on the surfaces of the hybrid films. A PS-100 four-point probe station (Lake Shore Cryotronics, Westerville, OH, USA) was used to measure sheet resistances with a Keithley 2400 source meter (Keithley, Cleveland, OH, USA).

### Fabrication and characterization of humidity sensors

The humidity sensors were prepared by patterning the PEDOT:PSS/PEG hybrid layer on the surface of photoinitiator (PI) activated PDMS with a specific photomask. After exposure to UV light with intensity of 20 mW cm^−2^ for 5 min, both the photomask and glass substrates were removed and the film was rinsed using DI water. Humidity tests were performed in a humidity control tank connected to nitrogen gas and a digital humidifier (7144, Air-O-Swiss, Widnau, Switzerland) to set different moisture levels. To analyze the device sensitivity and time response, an LCR meter (4284A, Agilent Technologies, Santa Clara, CA, USA) was used to detect capacitive changes with a sweep frequency of 1 kHz and an AC excitation voltage of 0.5 V. As a comparison, a commercial humidity sensor (HT110, PCE, Southampton, UK) was used, with 0.1% relative humidity (RH) resolution and <2 s time response.

## Results and discussion

### Photopatternability

**Figure 1:**
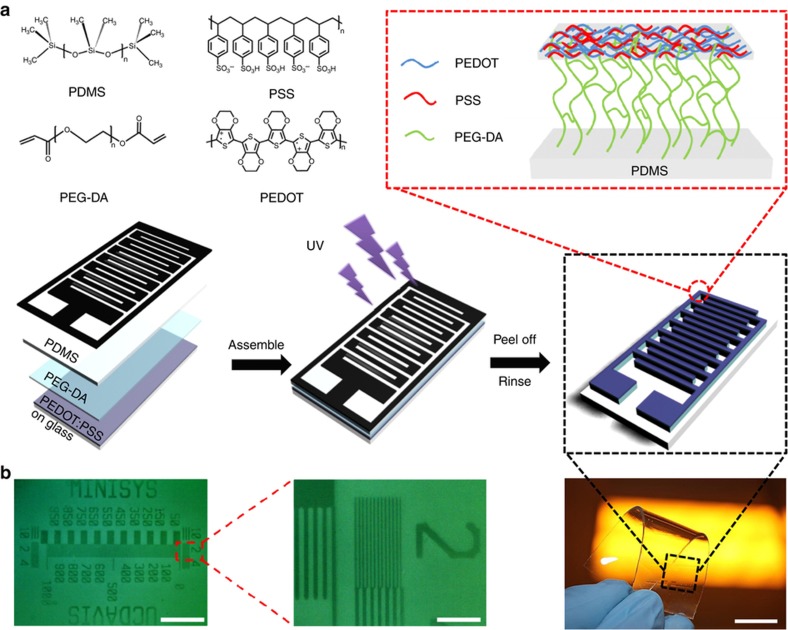
(**a**) Schematic illustration of the one-step photopatterning method to produce the PEDOT:PSS/PEG hybrid thin film, molecular structures of PEDOT:PSS, PEGDA, and PDMS, interactions between each layer, and an optical image of patterning the hybrid film on a PDMS substrate (scale bar, 1 cm). (**b**) Optical microscopy image of the patterned PEDOT:PSS/PEG hybrid film on PDMS (scale bar, 500 μm) and a magnified image showing feature resolution as small as 2 μm (scale bar, 50 μm).

Figure 1a shows the overall fabrication process of patterning the PEDOT:PSS/PEG hybrid film on a flexible surface (for example, a PDMS layer), by the one-step photolithographic method. Specifically, the PEDOT:PSS/PEG hybrid film was chemically linked to the PI-treated PDMS substrate with certain patterns under selective UV exposure through the photomask. After a washing step to remove PEGDA residues from the substrate, the patterned PEDOT:PSS/PEG hybrid film can be obtained on the flexible PDMS substrate, as shown in Figure 1b, with a feature resolution as small as 2 μm. Similar to the traditional photolithography technique, the feature resolution of PEDOT:PSS/PEG patterning was primarily determined by the optical wavelength of UV exposure and the separation distance. Therefore, the minimal resolution is within the micron range. To further improve the lithographic resolution, we can use a deep UV source (with a shorter wavelength) or reduce the thickness of the separation PDMS layer in addition to fine-tuning the exposure time. In brief, the entire processing method is rather straightforward and simple, consisting of assembling a photomask, an activated PDMS film, a photosensitive PEGDA layer, and a PEDOT:PSS-coated glass substrate, as well as UV exposure.

Currently, PEDOT:PSS processing is not completely compatible with conventional photoresists and developing procedures, as it is chemically sensitive to both acidic photoresists and alkaline developer solutions^34^. In the proposed photolithographic patterning of the PEDOT:PSS/PEG hybrid film, the one-step procedure is similar to traditional photolithography of negative photoresists, in which PEG behaves as a direct photosensitive matrix. This procedure has several advantages compared with the existing PEDOT:PSS patterning approaches, including a simple processing procedure, high resolution, and scalability to a large surface area^39^. It is worth noting that neither a protection layer nor a developer solution is needed in photopatterning, as the uncrosslinked PEGDA residues can be washed off by DI water, avoiding potential chemical damage to the sensitive PEDOT:PSS film. As a result, the moisture-stable PEDOT:PSS/PEG hybrid film also retains a high electrical conductivity of 500 S cm^−1^, which is equivalent to its ethylene glycol-doped counterpart^38^.

### Hybrid film integration

In the hybrid PEDOT:PSS thin film, PEGDA is functionalized with chemical linkages to both the conductive PEDOT:PSS film and the activated PDMS substrate, which becomes essential to form a chemically and mechanically stable hybrid thin film. Benzophenone serves as a PI to crosslink the PEGDA molecules, which first diffuses into the porous PDMS surface to form photoreactive areas. Wang *et al.*^40^ visualized the diffusion of benzophenone into a PDMS surface and polymerization inside the PDMS layer using a fluorescent dye when photopatterning poly (acrylic acid) on PDMS. Other groups have also reported a similar photoinduced grafting approach to patterning PEGDA onto a PDMS surface^41^. Considering a PEDOT:PSS conducting film containing a similar porous structure, it is reasonable to envision that the PEGDA solution can diffuse into the porous topology of PEDOT:PSS and crosslink the two chemicals together in a polymerization process^42^. Therefore, when the PEGDA solution is first flowed in between the photoreactive PDMS and PEDOT:PSS layers, the PEGDA molecules first diffuse into both porous PDMS and PEDOT:PSS structures and then form crosslinked networks under UV exposure.

**Figure 2:**
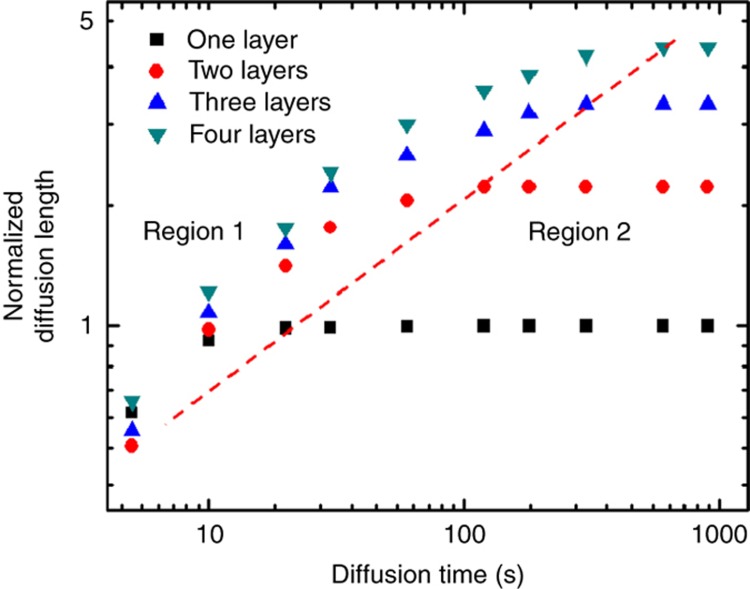
The amount of PEDOT:PSS that was transferred from glass to PDMS with different diffusion times was counted by testing the conductivity of PEDOT:PSS/PEG on the PDMS substrate. The normalized diffusion length was converted from normalized conductivity, which was directly proportional to the thickness of the PEDOT:PSS film. The red dashed line represents the relationship between the normalized diffusion length and saturated diffusion time; it also separates the graph into two regions, where region 1 is time-dependent and region 2 is thickness-dependent.

To further confirm our hypothesis of PEGDA diffusion into PEDOT:PSS, we have focused on our diffusion tests dedicated to crosslinking a different number of layers of PEDOT:PSS. According to the diffusion theory, PEGDA molecular diffusion into PEDOT:PSS layers can be expressed as

$$L\propto \sqrt{Dt}$$

where *L* and *t* stand for diffusion length and diffusion time, respectively, while *D* is the diffusion coefficient of PEGDA molecules in the PEDOT:PSS structure. It is expected that, with increments of diffusion time, PEGDA can further penetrate into the PEDOT:PSS structure. Subsequently, a thicker layer of PEDOT:PSS can be transferred onto the crosslinked PEG substrate, and, therefore, an increased electrical conductivity, which is supposedly proportional to the thickness of the PEDOT:PSS layer^39^, is expected to be measured from the hybrid PEDOT:PSS/PEG film. As previous publications stated^40–42^, the hybrid structure only polymerizes under UV exposure when the diffusion of PEGDA penetrates into the PEDOT:PSS layers. Thus, it is reasonable to approximate the PEGDA diffusion length as the thickness of the conductive PEDOT:PSS/PEG complex, which can be directly calculated from the measured film conductance. As the PEGDA-containing solution is introduced in between the PEDOT:PSS-coated substrate and the PI-activated PDMS layer, the diffusion time of PEGDA is considered to be the same as the processing time. After the UV exposure and substrate removal, the normalized diffusion length that converted from the electrical conductivities of the PEDOT:PSS/PEG hybrid films with various diffusion times and plotted in Figure 2, with a time-dependent region and a thickness-dependent region. According to the diffusion equation shown above, the diffusion length is supposedly proportional to the second power of the saturated diffusion time, which is indicated by the red dashed line with a correlation coefficient of 0.9968 calculated from the experimental results. In the time-dependent region, an increase of diffusion time leads to a rise in the PEGDA diffusion length. As a result, a thicker PEDOT:PSS film will be transferred onto the PDMS substrate. When PEGDA further diffuses, saturation occurred in the thickness-dependent region because the diffusion of PEGDA approaches the total thickness of the PEDOT:PSS layers. In this case, the diffusion length of the PEGDA will be determined by the total thickness of the PEDOT:PSS film instead of the time of diffusion. In brief, with an increase of the PEDOT:PSS film thickness, additional diffusion time is needed for PEGDA molecules to fully penetrate into the PEDOT:PSS structure.

### Moisture stability

**Figure 3:**
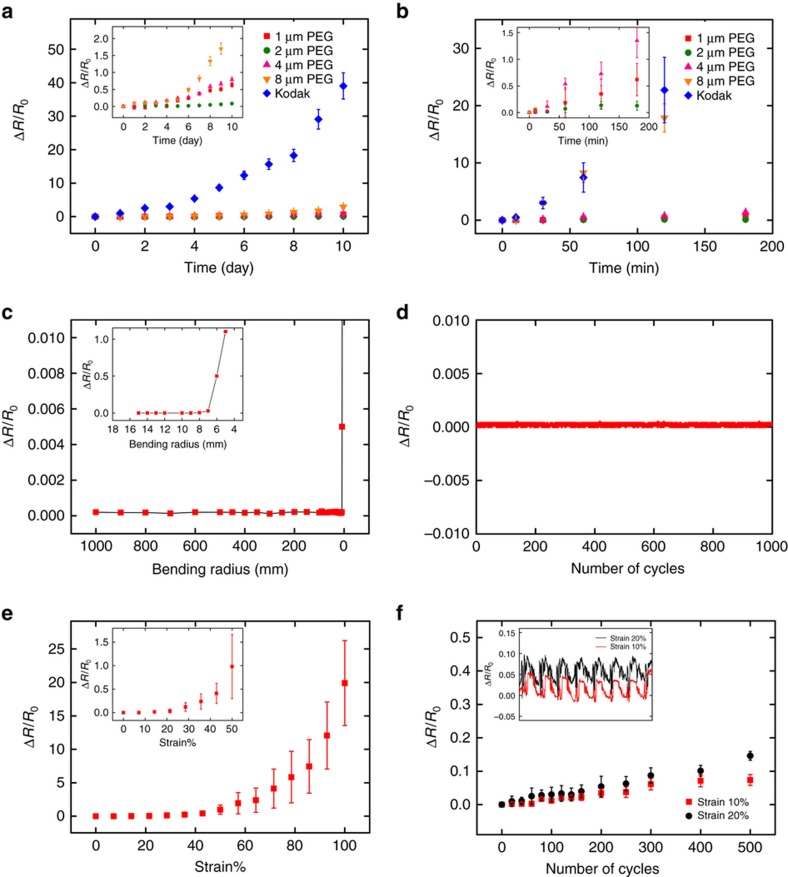
The relative resistance changes of the PEDOT:PSS/PEG hybrid films on PDMS. Moisture stability tests: (**a**) submerged in DI water for 10 days; and (**b**) ultrasonicated in DI water for 3 h. The fracture and degradation of the PEDOT:PSS that lead to the resistance increase were investigated by varying the PEG layer thickness, assuming that a thicker structure would lead to a larger surface strain and more interfacial cracks. Bending tests: (**c**) bending from flat to a 5 mm bending radius; and (**d**) repeated bending from flat to a 10-mm bending radius for 1000 cycles. Tensile tests: (**e**) external tensile strain from 0 to 100%; and (**f**) repeated stretching with 10% strain and 20% strain, respectively.

Figures 3a and b show the moisture stability of the PEDOT:PSS/PEG hybrid film. The changes of the resistance of the conductive hybrid films were assessed over a 10-day period, in which the PEG matrix had various thicknesses. Compared with a commercial PEDOT:PSS conducting film on polyester, the crosslinked PEDOT:PSS/PEG hybrid films exhibit relative stabilities in electrical resistance under DI water with marginal changes. In particular, the hybrid film with a 2-μm-thick PEG matrix illustrates the best moisture stability with <10% variation in resistance after 10 days of immersion in DI water, whereas the control group, the Kodak film, shows two orders of magnitude of change in the resistance measurement. We had taken the specimens out of the DI water and dried them before the conductance measurement, in which the hybrid film swells and shrinks repeatedly due to moisture absorption. As reported, PEG hydrogels have a lower fracture strain than PDMS^43^; thus, cracks and structure failure would occur at the PEDOT:PSS/PEG interface, especially when the hybrid film is under external mechanical forces. Due to hydrophobic nature of the PDMS substrate, interfacial strain between the PEG and PEDOT:PSS layers would increase gradually with the thickness of the PEG layer^44^. Therefore, it is reasonable to consider that the fracture and degradation of the PEDOT:PSS layer could be thickness-dependent; that is, a thicker hybrid layer corresponds to a larger surface strain and more interfacial cracks. In addition, a harsh-environment test for the moisture stability was also conducted under an ultrasonic bath for 3 h, which illustrated consistent results with the previous tests. The resistances of the crosslinked PEDOT:PSS/PEG hybrid films still showed significantly less increment in comparison with that of the commercial Kodak film. The highest electrical stability was demonstrated by the hybrid film with a 2-μm-thick PEG layer. Thus, in the following studies, the thickness of the PEG matrix is selected to be 2 μm in the hybrid films.

In addition, the optical transmittance of PEDOT:PSS/PEG hybrid film subjected to a 10-day water treatment was measured and is shown in Supplementary Figure S1. The PEDOT:PSS/PEG hybrid film retained high transparency (>80%) compared with PEDOT:PSS spin coated on glass, the PEDOT:PSS film (Kodak), and an untreated PEDOT:PSS/PEG hybrid film.

### Mechanical flexibility and stretchability

The PEDOT:PSS/PEG hybrid film also shows excellent mechanical flexibility and stretchability on flexible substrates (for example, PDMS), which can be of potential interest for wearable applications. Both mechanical bending and tensile tests were performed (Supplementary Figures S2a and b) and are summarized in Figures 3c–f, with the hybrid film on a 250-μm-thick PDMS substrate with a footprint of 2×5 cm^2^. The measurements were performed with a computer-controlled step motor that can precisely and continuously regulate the end-to-end length of the hybrid film, from which the bending radius and induced strain can be calculated (see the Supplementary Information for a detailed calculation). The electrical resistance of the hybrid film remained unaltered (less than a 0.1% variation) when bending over a surface with a radius curvature of 10 mm or greater. However, when the surface curvature approached 5 mm, the film resistance increased rapidly, possibly caused by a potential structural failure or film cracking. In addition, a cyclic bending test was also performed and is illustrated in Figure 3d, where the electrical resistance varied within 0.1% after 1000 cycles of bending from flat to a 10 mm radius. In brief, the PEDOT:PSS/PEG hybrid film exhibited satisfactory mechanical flexibility results. Additional tensile tests were carried out. Figure 3e shows the changes of resistance when the film was extended from 0 to 100% strain at a constant speed of 1 mm s^−1^. Before the conducting hybrid film was stretched to 50% strain, the resistance changed gradually. Whereas, with extensive stretching from 50% strain and above, the electrical resistance experienced a drastic change and could reach ~20 times its initial value at 100% strain. The scanning electron microscopic image revealed the structural alternation beyond 50% strain, where apparent film cracking can be observed (Supplementary Figure S2c). Figure 3f illustrates the evolution of the film conductance over 500 cyclic stretching tests with 10 and 20% strain. With 10% repetitive strain loading, no obvious film buckles or cracks were shown on the surface, and the measured resistance remained within 10% of its original value. With 20% repetitive strain loading, the surface of the film started showing both non-periodic and periodic buckles (Supplementary Figure S2d) and the electrical resistance kept climbing and reached 15% change after 500 cycles.

### Moisture sensitivity

**Figure 4:**
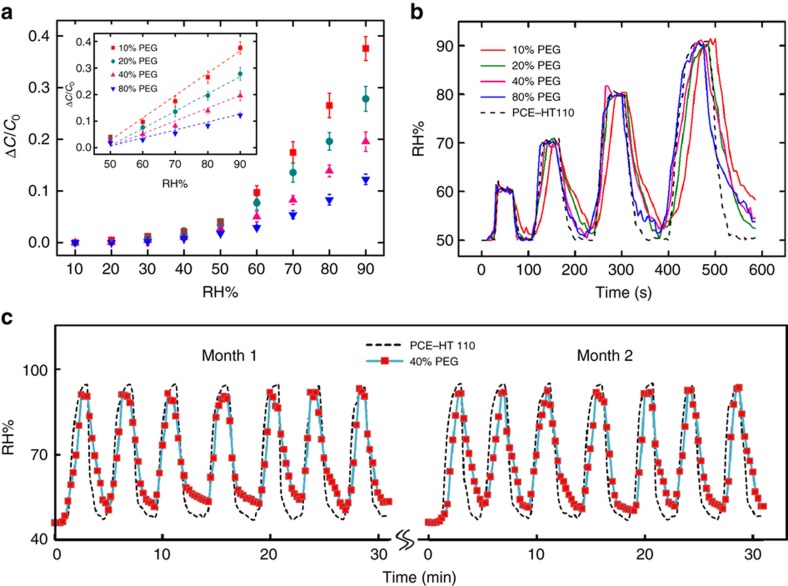
(**a**) Measured capacitive changes of the humidity sensors under different RH levels (dots) with theoretical predictions (dashed lines). The sensors were fabricated with different PEG contents (10, 20, 40, and 80%) during the polymerization process, and five identical devices were tested for each PEG ratio. (**b**) Sensor responses to environmental humidity change with different PEG contents (10, 20, 40, and 80%). RH was changed between 50 and 60%, then 50 and 70%, 50 and 80%, and finally 50 and 90%, consecutively. (**c**) Cyclic humidity tests over a two-month period using the sensor with 40% PEG content. RH was controlled, changing between RH 50% and RH 90%, repeatedly.

To build a humidity-sensing device, we utilized the moisture-responsive nature of the PEG matrix, since water molecules can diffuse through its porous network. When water vapor is in contact with the hybrid film, the water molecules can be physically absorbed by or condensed onto the film surface (Supplementary Figure S3a), while the contained water contents on the surface would continue evaporating into the atmosphere until an equilibrium is established^45^. The alternation of moisture content in the matrix leads to the change in the permittivity of the PEG hydrogel (Supplementary Figure S3b), which can be detected as a capacitive shift in a single-sided capacitor configuration^46^. Therefore, a higher environmental humidity level would result in a higher water content in PEG matrix and, herein, a greater permittivity and capacitance change^47^. Using a classic fringe capacitor model^46^, the device capacitance of the moisture sensor can be expressed by humidity-induced permittivity variation as

$$C\;=\;\frac{(\;N\;+\;1\;)C_{I}}{2}\varepsilon_{0}L\left({\;\varepsilon }_{\mathrm{air}}C\left(\infty \right){\left(\frac{1}{\varepsilon_{p}C\left(h\right)}\;+\;\frac{1}{\varepsilon_{s}}\left(\frac{1}{C\left(h\;\right)}-\;\frac{1}{C\left(\;\infty \;\right)}\;\right)\;\right)}^{\;-\;1}\;\right)$$

where *C*_*I*_ stands for the half capacitance between electrodes and *N* is the number of electrode pairs. *e*_air_, *e*_p_, and *e*_s_ indicate the relative permittivity values of air, the PEG layer, and the PDMS substrate, respectively. Moreover, *C*(*∞*) and *C*(*h*) are considered as the geometric capacitance values of an infinite layer and a PEG layer of *h* thickness, respectively (see the Supplementary Information for a detailed calculation). In the PEDOT:PSS/PEG hybrid film, the porosity of the PEG matrix has an important role in determining the permittivity change^36^. To investigate such an influence, we have fabricated and prepared capacitive humidity-sensing devices with various levels of PEG contents (10, 20, 40, and 80%), which have been characterized in a controlled humidity chamber monitored by a commercial humidity sensor (PCE-HT 110). Figure 4a summarizes the calculated and measured capacitive changes versus RH ranging from RH 10% to RH 90%. The experimental results (data points) and theoretical predications (dashed lines) show a high correlation in the high RH range. Specifically, the sensors with lower PEG contents behave more responsively to the moisture than those with higher PEG contents (0.0085/RH%, 0.0065/RH%, 0.0045/RH%, and 0.0027/RH%, respectively). This is highly consistent with the fact that a higher porosity level with larger pore sizes is present in a PEG matrix with lower PEG content, and therefore, more moisture can be absorbed into the porous matrix^48,49^. Due to the high permittivity nature of the water molecules, a more detectable change in the permittivity in such a matrix can be observed under the same humidity level.

As another key parameter, response time was also investigated for the humidity sensors with different PEG contents. The humidity level was adjusted between 50 and 60%, then 50 and 70%, 50 and 80%, and finally 50 and 90%, consecutively. The changes in the capacitance for each humidity sensor were recorded and are converted to the RH level in Figure 4b. As indicated by the measurement results, the response time of our PEDOT:PSS/PEG humidity sensor was comparable with that of the commercial one (PCE, HT-110), which was claimed to be responsive to a humidity change in <2 s (Ref. 50). In addition, the influence of the thickness with the PEG matrix to the response time was also studied, and the results are summarized in Supplementary Figure S4. No appreciable difference in response time was observed, which suggests that either the diffusion of water molecules was rapid or the surface absorption process dominated the response time of the humidity sensor (that is, diffusion time is negligible)^45,51^.

To further explore the repeatability of this humidity sensor, cyclic humidity tests were conducted over a 2-month period, where RH was adjusted between 50 and 90% periodically. Considering the influences of the PEG matrix in humidity sensing, we have used the sensors with 40% PEG content in a 2-μm-thick layer. Figure 4c shows the humidity measurements from our capacitive humidity sensor compared with the commercial unit, both of which followed the same trend. Moreover, after 1 month of testing, the humidity sensor showed a nearly identical performance without appreciable sensor degradation or signal variation observed.

### Demonstrations

**Figure 5:**
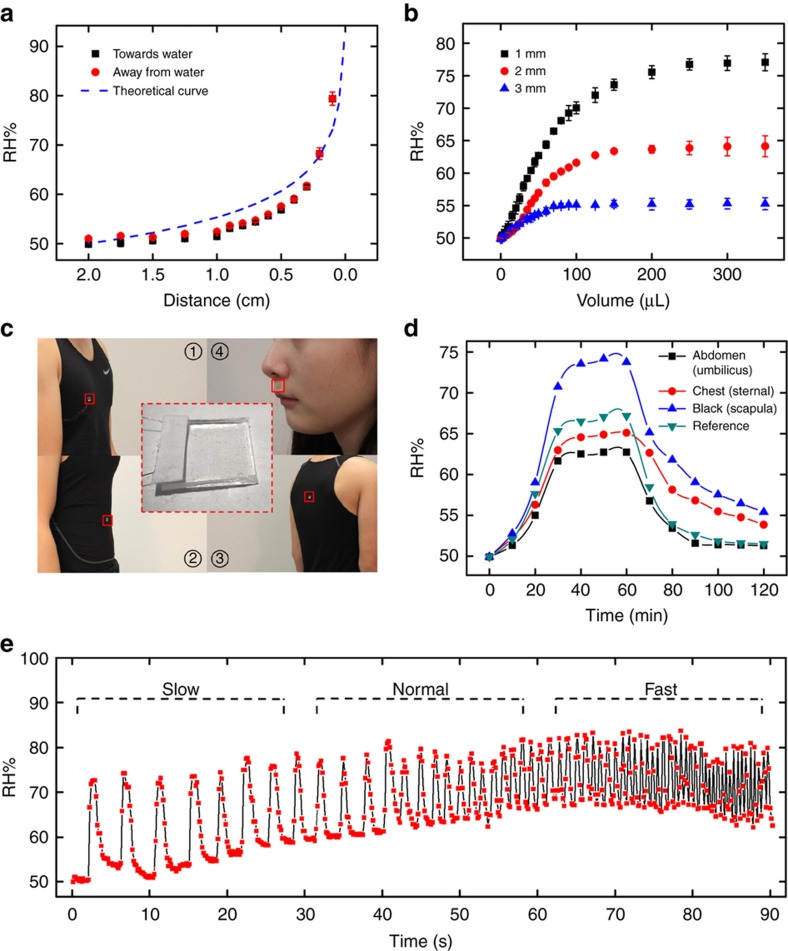
(**a**) Changes of RH, as the PEDOT:PSS/PEG hybrid humidity sensor moved towards and away from the water surface. The theoretical curve was plotted with a dashed line. (**b**) Changes of RH, as water was introduced onto fabric constantly. Distances between sensor and fabric were fixed at 1, 2, and 3 mm. (**c**) Pictures of sensors on clothes (abdomen, chest, and back) and skin (under nose). (**d**) Changes of RH calculated from capacitive changes of the sensors attached on abdomen (umbilicus), chest (sternal), and back (scapula), respectively, with a comparison to the reference that calculated from changes in the weight of the clothes with sweat. (**e**) Responses of the sensor at different breathing rates.

With increasing awareness and demands regarding personalized health care, wearable sensors have been extensively investigated in recent years, for which electrical conductance, mechanical flexibility, chemical stability, optical transparency, and biocompatibility are all required^52–56^. The conductive PEDOT:PSS film has proven to be a candidate material for such wearable applications^57,58^. We have applied the capacitive humidity sensors made from the PEDOT:PSS/PEG hybrid film as demonstrations in various scenarios, benefiting from its one-step photopatterning, excellent moisture stability and sensitivity, and satisfactory mechanical flexibility. Figure 5a shows that the humidity sensor has been utilized as a measurement device to profile the humidity level from a water surface. The measured RH, calculated from the change of capacitance, increases with the approach to the water surface, as expected, which fits very well with the theoretical predications (a detailed calculation can be found in the Supplementary Information)^59,60^. The test continues as the humidity sensor moves away from the water surface, for which the measurement results closely follow the readings from the approaching process and show a negligible hysteretic effect overall. This again confirms the sensing accuracy and consistency of the PEDOT:PSS/PEG devices.

To further extend this flexible humidity sensor to a fabric surface, we have targeted it for wearable continuous perspiration measurement. First, we set up a calibration test of the humidity sensors at different distances from the surface of a fabric (92% polyester and 8% spandex), which is wetted by gradually introducing DI water to the water-absorbing matrix. As plotted in Figure 5b, the measured RH by the sensor exhibits a gradual increase at the beginning (during the wetting process) and eventually reaches an equilibrium as the fabric is completely soaked. On the basis of the separation distance between the soaking fabric and the sensor, a variety of levels of humidity have been measured, such as 55% at 1 mm separation, 64% at 2 mm separation, and 77% at 3 mm separation, respectively. Moreover, we can determine the total volume being absorbed by the fabric using this measurement curve, from which a perspiration rate can be calculated. In the following wearable humidity measurement, the PEDOT:PSS/PEG sensing devices were prepared and attached to the external surface of clothes (made of the same materials as the calibration fabric) on the abdomen, chest, and back of a healthy adult volunteer (Figure 5c).

The changes in capacitance for each sensor were tracked and converted to RH every 10 min for 1 h continuous jogging on a treadmill followed by 1 h of rest by the volunteer. The calculated perspiration rates from the capacitive measurements for different parts of the body have been plotted and are summarized in Figure 5d. As a reference, we have included the weight change measurement of the clothes as well, all of which have shown the same trend. The physiological literature states that the back produces more sweat than both the abdomen and chest, which is consistent with our observation^61^.

Finally, a breath rate measurement was performed, which can be of interest for both consumer applications (for example, exercise monitoring)^62^ and medical diagnosis (for example, detection of sleep apnea)^63^. To this end, we attached the humidity sensor onto the skin right underneath the nose. With the exhale, the humidity above the sensor will increase, as the exhalant is highly moisturized by our lungs. During the inhale, the external flow from the environment will decrease the moisture level around the sensor. As a result, a cyclic pattern of humidity changes can be used as a simple method to calculate the breath rate. Its non-obstructive nature allows continuous *in situ* monitoring as a simple wearable device. Figure 5e shows the recorded breath signals, from which three breathing modes have been detected, that is, slow breathing mode (rest/sleep ~0.2 Hz), normal breathing mode (~0.5 Hz), and fast mode (exercise/stressed ~2 Hz). As confirmed by the experimental results, our humidity sensor can rapidly respond to slow and fast evolving humidity changes and track the breath rate with sufficient bandwidth, which can serve as a powerful alternative to the current standard for breath rate measurement^64^.

In summary, for the first time, we have developed a PEDOT:PSS/PEG hybrid thin film structure, which can be photopatterned in one step with high lithographic resolution, excellent moisture stability and sensitivity, and satisfactory mechanically flexibility. An interesting UV-enabled polymerization process is introduced in which both the PEDOT:PSS conductive layer and porous PI-activated PDMS substrate are directly crosslinked with the PEGDA solution and form strong chemical linkages on the hybrid film structure. Consequently, the hybrid thin-film structure inherits high electrical conductivity from PEDOT:PSS, excellent mechanical flexibility from PDMS and becomes moisture responsive from the PEG matrix. Given this powerful combination, we have applied the thin-film hybrid as a capacitive humidity sensor, which can be utilized in a variety of ways, including emerging wearable perspiration measurements and breath rate monitoring. PEG-based materials have been proven to be tissue-biocompatible and highly stable with temperature and pH, making them ideal candidates for drug delivery and tissue engineering^65,66^. Recent publications have also suggested biocompatibility for PEDOT:PSS both *in vivo* and *in vitro*, in which PEDOT:PSS has been applied as an electrode material at neural interfaces and as scaffolds for cell adhesion and proliferation, respectively^9,11,67^. Therefore, PEDOT:PSS/PEG hybrid thin films have great potential to be further utilized in bio-hybrid devices as a biocompatible material for applications, such as tissue scaffold and biosignal recording, as well as in electrical stimulations for cell migration, growth, and differentiation, where biocompatibility, biostability, and structural patternability are all required.
