# Supplementary material for: Photopatternable PEDOT:PSS/PEG hybrid thin film with moisture stability and sensitivity
Source: Microsyst Nanoeng. 2017 Apr 10;3:17004. doi: 10.1038/micronano.2017.4 (PMC6445012; doi:10.1038/micronano.2017.4)
Supplement: Supplementary Information [file micronano20174-s1.pdf]

Supplementary file

Photopatternable PEDOT:PSS/PEG hybrid thin film with moisture stability and sensitivity

Zijie Zhu[1,2], Gaomai Yang[1], Ruya Li[1,2] and Tingrui Pan[1]

Microsystems & Nanoengineering (2017) **3,** 17004; doi:10.1038/micronano.2017.4; Published online: 10 April 2017

THEORETICAL CALCULATION OF BENDING RADIUS AND INDUCED STRAIN

As shown in Supplementary Figure S2, the computer-controlled step motor can control the end-to-end length (*L-dL*) of the hybrid film. Therefore, we can use the experimentally measured distant change to calculate the bending radius (*r*) and its induced strain (*ε*), using the classic bending theory as outlined below[1]:

$$r = \frac{L}{2\pi\sqrt{\frac{dL}{L} \quad \frac{\pi^2 h^2}{12L^2}}}$$

$$\varepsilon = \frac{h}{2r}$$

where *L* is the initial film length, *dL* is the compressed distance, and *h* is the thickness of the hybrid film. Using the equations above, the strain experienced by PEDOT:PSS film is estimated at 1.25% given a bending radius of 10 mm.

THEORETICAL CALCULATION OF DEVICE SENSITIVITY

PEG has a strong capacity to absorb water from humid environment, and the amount of water that can be absorbed is determined by the open pores volume, radius size, and pore distribution. To estimate change of capacitance under different humidity level in theory, different PEGDA concentration of water solutions were prepared and PEG hydrogel films with different pore sizes were formed under UV exposure. After fully dried in 80 °C oven for 1 h, PEG films were put into humidity control tank, which is controlled by nitrogen gas and Air-O-Swiss 7144 humidifier. All PEG films were maintained under each relative humidity environment for 30 min and then weighed the change of mass. The fractional volume of water absorbed in each PEG films under different relative humidity was plotted in Supplementary Figure S3a. Since water has relatively high dielectric constant (~80) compared with PEG (~10), dielectric constant of PEG film will increase after water sorption, which can be expressed as:

$$\varepsilon_{wet} = \left[\gamma\left(\varepsilon_{water}^{\frac{1}{3}} \quad \varepsilon_{dry}^{\frac{1}{3}}\right) + \varepsilon_{dry}^{\frac{1}{3}}\right]^3$$

And then

$$\frac{\Delta\varepsilon}{\varepsilon_0} = \frac{\varepsilon_{wet} \quad \varepsilon_{dry}}{\varepsilon_{dry}}$$

where γ is the fractional volume of water that absorbed in the PEG film, ε_{wet} and ε_{dry} are dielectric constants of wet and dry PEG film, respectively[2]. According to the relationship between dielectric constant and water uptake fraction, the change of dielectric constant of PEG versus relative humidity was plotted in Supplementary Figure S4b.

Considering dielectric constant differences between different components and edge effects, the capacitance of the fringe capacitive humidity sensor can be expressed as[2]:

$$C = (N \quad 3)\frac{C_I}{2} + \frac{2C_I C_E}{C_I + C_E}, \qquad N > 3$$

$$C_I = \varepsilon_0 L\left(\varepsilon_{air} C(\infty)\left(\frac{1}{\varepsilon_p C(h)} + \frac{1}{\varepsilon_s}\left(\frac{1}{C(h)} \quad \frac{1}{C(\infty)}\right)\right) \quad ^1\right)$$

$$C_E = \varepsilon_0 L\left(\varepsilon_{air} C'(\infty)\left(\frac{1}{\varepsilon_p C'(h)} + \frac{1}{\varepsilon_s}\left(\frac{1}{C'(h)} \quad \frac{1}{C'(\infty)}\right)\right) \quad ^1\right)$$

where C_I is the half capacitance between one interior electrode and ground, C_E is the capacitance between one outer electrode and ground, and *N* is the number of electrodes. ε_{air}, ε_p, and ε_s, are relative dielectric constant of air, PEG layer, and PDMS substrate layer, respectively. $C(\infty)$ is the geometric capacitance of an infinite layer and $C(h)$ is the geometric capacitance of the PEG layer with

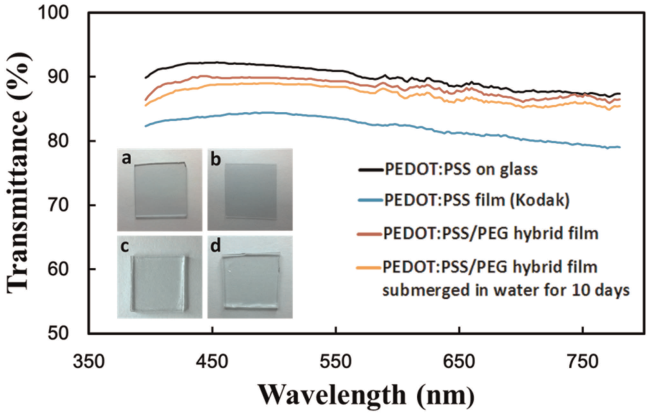

Figure S1 Transmittance measurement. Optical transmittance and photos of (a) PEDOT:PSS film spin-coated on glass. (b) Commercial PEDOT:PSS film on PET (Kodak). (c) PEDOT:PSS/PEG hybrid film on PDMS before water treatment. (d) PEDOT:PSS/PEG hybrid film on PDMS after 10-day water treatment.

[1]Micro-Nano Innovations (MiNI) Laboratory, Department of Biomedical Engineering, The University of California, Davis 95616, USA and [2]Department of Electrical and Computer Engineering, The University of California, Davis 95616, USA
Correspondence: Tingrui Pan (tingrui@ucdavis.edu)

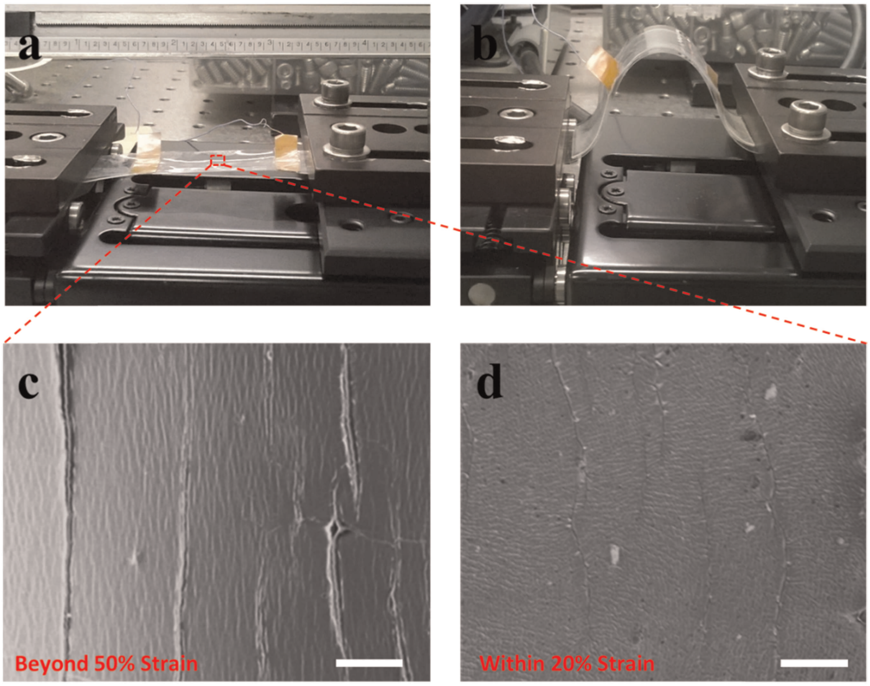

Figure S2 Tensile and bending tests. Measurement set-up for (**a**) tensile and (**b**) bending tests and SEM images of the surface of PEDOT:PSS/PEG hybrid film on PDMS after (**c**) stretched beyond 50% strain (scale bar: 50 μm), (**d**) stretched within 20% strain (scale bar: 10 μm).

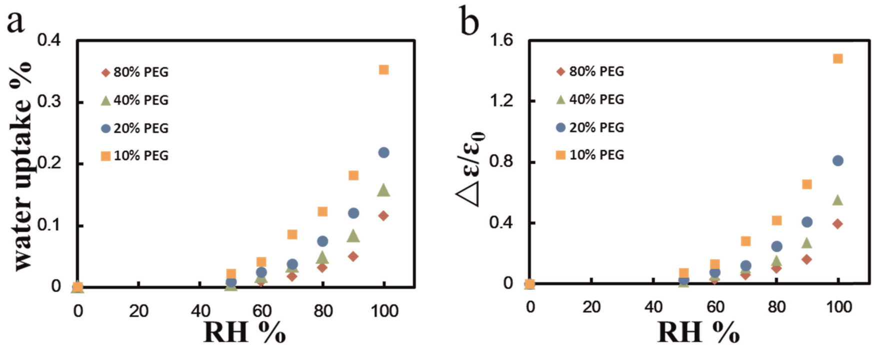

Figure S3 (**a**) Fractional volume of water absorbed in various PEG film under different humidity environment. (**b**) Theoretically calculation of PEG dielectric constant change under different humidity environment.

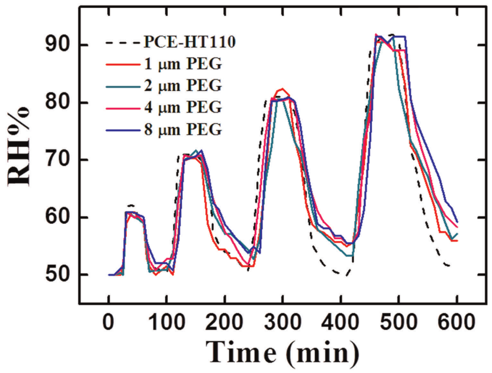

Figure S4 PEG thickness effect on response time. Sensor reponses to humidity change with different PEG thicknesses.

thickness h. To simplify calculation, edge effect was neglected and equations above can be expressed as:

$$C = \frac{(N+1)}{2} C_l$$
$$= \frac{(N+1)}{2} \varepsilon_0 L \varepsilon_{air} C(\infty) \left(\frac{1}{\varepsilon_p C(h)} + \frac{1}{\varepsilon_s} \left(\frac{1}{C(h)} \quad \frac{1}{C(\infty)} \right) \right)^{1}$$

Since we only care about the change of dielectric constant in PEG and the change in air and substrate can be neglected, then the change of capacitance can be simplified as:

$$\frac{\Delta C}{C_0} = \frac{\frac{\varepsilon_{wet}}{\varepsilon_{wet}+\varepsilon_s} \quad \frac{\varepsilon_{dry}}{\varepsilon_{dry}+\varepsilon_s}}{\frac{\varepsilon_{dry}}{\varepsilon_{dry}+\varepsilon_s}} \cong \frac{\frac{\Delta\varepsilon}{\varepsilon_0}}{\frac{\Delta\varepsilon}{\varepsilon_0} \quad 1}$$

where ε_s is dielectric of PDMS substrate (~2.5).

THEORETICAL CALCULATION OF DEVICE SENSITIVITY

According to Montgomery's work[3], the verticall gradient of water vapor can be expressed as:

$$\frac{\mathrm{d}P}{\mathrm{d}\ln z} = \frac{P_b \quad P_a}{\ln b/a}$$

And

$$\Gamma \equiv \frac{P_b \quad P_a}{P_s \quad P_a}\frac{1}{\ln b/a}$$

where P_b, P_a, and P_s are vapor pressure at the height a, b, and at the surface, respectively. For a smooth surface, assuming that the vertical transfer of water vaper is independent of height, the evaporation coefficient can be calculated by[4]:

$$\Gamma = \left(\frac{\lambda \nu k_0}{\kappa} + \ln\frac{k_0 \gamma_0 W_0 a}{\kappa}\right)^{\quad 1}$$

where λ is a numerical factor, κ is the kinematic coefficienct of diffusin of vapor in air, v is the kinematic viscosity of the air, k_0 is von Karman's constant, γ_0 is the resistance coefficient, and W_0 is the wind velocity at the height a, in which way, the change of RH is direcly proportional to $-\ln(b/a)$.

REFERENCES

1 Park SI, Ahn JH, Feng X *et al.* Theoretical and experimental studies of bending of inorganic electronic materials on plastic substrates. *Advanced Functional Materials* 2008; **18**: 2673–2684.

2 Shibata H, Ito M, Asakursa M *et al.* A digital hygrometer using a polyimide film relative humidity sensor. *IEEE Transactions on Instrumentation and Measurement* 1996; **45**: 564–569.

3 Igreja R, Dias CJ. Analytical evaluation of the interdigital electrodes capacitance for a multi-layered structure. *Sensors and Actuators A*: *Physical* 2004; **112**: 291–301.

4 Montgomery RB. Observations of vertical humidity distribution above the ocean surface and their relation to evaporation. *Paper in Physical Oceanography and Meteorology* 1940; **7**: 30.
